# Structural Comparison of Two CSPG-Binding DBL Domains from the VAR2CSA Protein Important in Malaria during Pregnancy

**DOI:** 10.1016/j.jmb.2009.08.027

**Published:** 2009-10-16

**Authors:** Pongsak Khunrae, Judith M.D. Philip, Duncan R. Bull, Matthew K. Higgins

**Affiliations:** Department of Biochemistry, University of Cambridge, 80, Tennis Court Road, Cambridge CB2 1GA, UK

**Keywords:** CSPG, chondroitin sulfate proteoglycan, CSA, chondroitin sulfate A, DBL, Duffy binding-like, PEG, polyethylene glycol, TEV, tobacco etch virus, MAD, multiple-wavelength anomalous dispersion, malaria, pregnancy, PfEMP1, VAR2CSA, chondroitin sulfate

## Abstract

Severe malaria during pregnancy is associated with accumulation of parasite-infected erythrocytes in the placenta due to interactions between VAR2CSA protein, expressed on the surface of infected-erythrocytes, and placental chondroitin sulfate proteoglycans (CSPG). VAR2CSA contains multiple CSPG-binding domains, including DBL3X and DBL6ɛ. Previous structural studies of DBL3X suggested CSPG to bind to a positively charged patch and sulfate-binding site on the concave surface of the domain. Here we present the structure of the DBL6ɛ domain from VAR2CSA. This domain displays the same overall architecture and secondary structure as that of DBL3X but differs in loop structures, disulfide bond positions and surface charge distribution. In particular, despite binding to CSPG, DBL6ɛ lacks the key features of the CSPG-binding site of DBL3X. Instead DBL6ɛ binds to CSPG through a positively charged surface on the distal side of subdomain 2 that is exposed in intact VAR2CSA on the erythrocyte surface. Finally, unlike intact VAR2CSA, both DBL3X and DBL6ɛ bind to various carbohydrates, with greatest affinity for ligands with high sulfation and negative charge. These studies provide further insight into the structure of DBL domains and suggest a model for the role of individual domains in CSPG binding by VAR2CSA in placental malaria.

## Introduction

Malaria is the most deadly parasitic disease of mankind, causing 500 million serious cases and in excess of 1 million deaths each year.^1^ While people from endemic countries develop partial immunity after multiple disease episodes, pregnant women are particularly susceptible. Central to malaria during pregnancy is accumulation of infected erythrocytes in the intervillous space of the placenta. The placenta provides an environment in which the parasite can flourish, leading to maternal anemia and causing placental inflammation and impaired blood flow to the developing child.^2–4^ Malaria during pregnancy is implicated in the death or underweight birth of many children and kills an estimated 75,000–200,000 fetuses each year.^5^

The intervillous space of the placenta contains chondroitin sulfate proteoglycans (CSPGs). The carbohydrate component of CSPG, chondroitin sulfate A (CSA), acts as the primary placental receptor for infected erythrocytes. Erythrocytes from pregnant women bind to CSA, while those from malaria-infected men or children do not. In addition, soluble CSA inhibits the binding of infected erythrocytes to placental tissue.^6^ These interactions appear to be highly specific. While 4-sulfated chondroitin structures inhibit the adhesion of infected red blood cells to placental CSPG, other glycosaminoglycan carbohydrates, including chondroitin sulfates B and C, heparin and hyaluronic acid, do not.^7–9^ In addition, the percentage of 4-sulfation alters the ability of carbohydrate to bind to infected erythrocytes, with a combination of unsulfated and 4-sulfated disaccharides providing the highest efficacy.^8–11^

The CSA receptor on infected erythrocytes is a PfEMP1 protein. PfEMP1s are adhesive proteins^12–14^ containing multiple discrete extracellular domains, falling mostly into two families, the cysteine-rich interdomain region and the Duffy binding-like (DBL) domains.^15^ These have important binding properties, interacting with a variety of human receptors.^16–18^ While several DBL domains from different PfEMP1 proteins bind to CSA, VAR2CSA is the primary CSA receptor in placental malaria.^5,19–21^ VAR2CSA shows upregulated expression in placental parasite isolates,^22^ binds to CSA with a specificity similar to that of infected erythrocytes^23^ and contains multiple DBL domains that can bind to CSPG when isolated.^24–26^ Immunity that develops following multiple pregnancies is associated with antibodies that bind to VAR2CSA protein^21^ and block CSA binding.^27–29^ Indeed, antibodies raised against the individual domains of VAR2CSA reduce the binding of infected erythrocytes to CSA.^30,31^ This suggests that the interaction between VAR2CSA and CSPG is the principal target for development of therapeutics to target placental malaria.

Two recent articles describe structures of the DBL3X domain of VAR2CSA, revealing an α-helical core with extensive loop insertions.^32,33^ One structure shows that a loop on the concave face of the domain becomes ordered in the presence of sulfate ions or disaccharide, completing the major patch of positive charge on the domain surface.^32^ However, only the sulfate ion of the disaccharide can be observed, suggesting that other parts are disordered. A second structure of DBL3X, crystallised with longer CSA oligosaccharides, showed electron density close to this positively charged patch, but this density was not well-defined or continuous, preventing model building.^33^ This surface was suggested by both studies to form the CSPG-binding site, and mutations of two lysine residues in the positively charged patch (K1507 and K1510) reduced binding of the domain to CSPG.^33^

These studies raised many questions about the role of DBL domains in binding to CSPG. Do different DBL domains bind to their ligand through a conserved site? Do individual DBL domains show the same ligand-binding specificity as that of intact VAR2CSA? Does VAR2CSA consist of a series of independent, specific ligand-binding domains or do domains combine to form a specific binding pocket? To provide insight into some of these questions, we determined the structure of the DBL6ɛ domain of VAR2CSA and used surface plasmon resonance measurements to investigate the CSPG-binding sites and ligand specificity of the DBL6ɛ and DBL3X domains.

## Results

### The structure of the DBL6ɛ domain of VAR2CSA

**Fig. 1:**
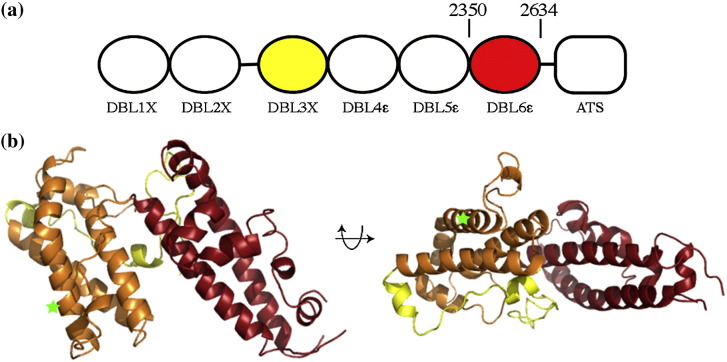
The structure of the DBL6ɛ domain of VAR2CSA. (a) The VAR2CSA protein consists of six extracellular DBL domains, followed by a single transmembrane helix and a cytoplasmic acidic terminal sequence (ATS). (b) Orthogonal views of the DBL6ɛ domain (residues 2350–2634) with subdomain 1 in yellow, subdomain 2 in orange and subdomain 3 in red. Green stars represent the position of residue 2480.

Using a bacterial expression system, we expressed the DBL6ɛ domain of VAR2CSA (residues 2333–2634) from the 3D7 strain of *Plasmodium falciparum*. Structure-based alignment of this sequence to the structure of DBL3X^32^ suggests that C2480 does not form a disulfide bond with another residue from DBL6ɛ. We expressed both the DBL6ɛ domain and the C2480S mutant and were able to crystallise C2480S. The structure was determined using multiple-wavelength dispersion data from a selenomethionine derivative and was refined to 3.0 Å resolution (Fig. 1).

**Fig. 2:**
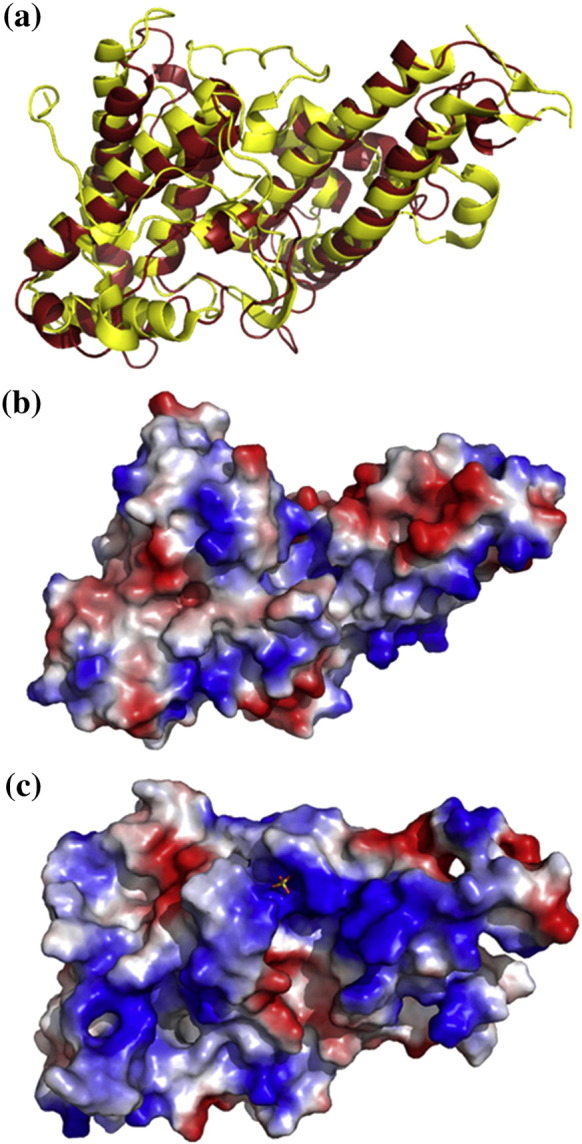
Comparison of the structures of DBL3X and DBL6ɛ. (a) Structural alignment of DBL3X [Protein Data Bank (PDB) code 3BQK] (yellow) with DBL6ɛ (red) showing the conserved α-helical scaffold and shorter loop structures of DBL6ɛ. Electrostatic surface representations of (b) DBL6ɛ and (c) DBL3X. Positively charged residues (+ 1.8) are blue, whereas negatively charged residues (− 1.8) are red.

DBL6ɛ shares a similar basic fold with other DBL domains^32–35^ containing a “boomerang”-shaped α-helical core that aligns to the core of the DBL3X structure with a root-mean-square deviation of 1.6 Å (Fig. 2a). However, there are many differences between DBL3X and DBL6ɛ. DBL6ɛ is shorter (302 residues compared with 359 for DBL3X) due to more compact loop structures and a shorter subdomain 1 (Fig. 2a). DBL6ɛ also contains fewer disulfide bonds than other DBL domain structures, lacking three of the bonds that stabilise the first two subdomains of DBL3X. In addition, the surface charge distribution of the two domains differs significantly, with the positively charged patch on the concave surface of DBL3X (Fig. 2c), predicted to form the CSPG-binding site,^32,33^ lacking in DBL6ɛ (Fig. 2b).

Subdomain 1 of DBL6ɛ consists of residues 2333–2390 with residues 2333–2348 disordered in the crystal. This subdomain mostly lacks secondary structure and, unlike this region in other known DBL domains, does not contain internal disulfide bonds. Subdomain 2 (2391–2506) contains four helices with several short loops. While all other current DBL domain structures contain a disulfide bond linking the second helix of subdomain 2 to the linker that connects subdomains 2 and 3, this is not present in DBL6ɛ, showing that this bond is not essential for the DBL domain fold. Indeed, there are no internal disulfide bonds in subdomain 2. The loops in this subdomain are significantly shorter than those of DBL3X and the loop that forms the sulfate-binding site in the predicted CSPG-binding surface of DBL3X (residues 1325–1341) is missing in DBL6ɛ.

Subdomain 3 (2507–2634) contains five disulfide bonds and consists of two long α-helices with a third strand completing the bundle. The distal part of the bundle is stabilised by three disulfide bonds (C2534–C2551, C2539–C2633 and C2555–C2631) and contains a single disordered loop. The remaining two disulfide bonds stabilise the third strand, with C2520–C2604 linking the third strand to the first helix of the subdomain, while C2598–C2602 stabilises the end of a loop extension in the third strand. The C2598–C2602 disulfide bond has not been observed in other DBL domain structures. Indeed, while the structurally characterised DBL domains contain as many as eight disulfide bonds, only the three at the bottom of subdomain 3 are observed in all current structures.

Comparison of the structures of DBL3X and DBL6ɛ shows differences in loop length, disulfide bonding pattern and surface charge distribution. Indeed, the features proposed to form the CSPG-binding site of DBL3X, the sulfate-binding loop and the positively charged surface, are absent in DBL6ɛ, agreeing with a recent prediction based on molecular modeling.^36^ To characterise the CSPG-binding surface of DBL6ɛ, crystals were grown in the presence of a variety of fragments of CSPG and in the presence of sulfate ions. However, no clear additional density due to ligand could be observed after structure determination. We therefore developed a surface plasmon resonance assay to study CSPG binding and used mutagenesis to map the residues involved in the CSPG-binding sites of DBL3X and DBL6ɛ.

### The CSPG-binding surface of DBL3X

To map residues involved in CSPG binding we covalently coupled human placental CSPG to a Biacore chip. To verify the assay, we first tested the ability of DBL3X to bind to the chip surface, measuring its binding to a CSPG-coated surface and subtracted the binding to a control surface that lacked the ligand. This showed clear binding of DBL3X to CSPG. As a control, we also tested the ability of DBL1X and DBL4ɛ of VAR2CSA to bind to CSPG. Neither DBL1X nor DBL4ɛ showed any observable binding to CSPG at concentrations of protein up to 100 μM (data not shown). Indeed, in contrast to DBL3X, both DBL1X and DBL4ɛ bound more to the control surface than to the CSPG-coated surface.

**Fig. 3:**
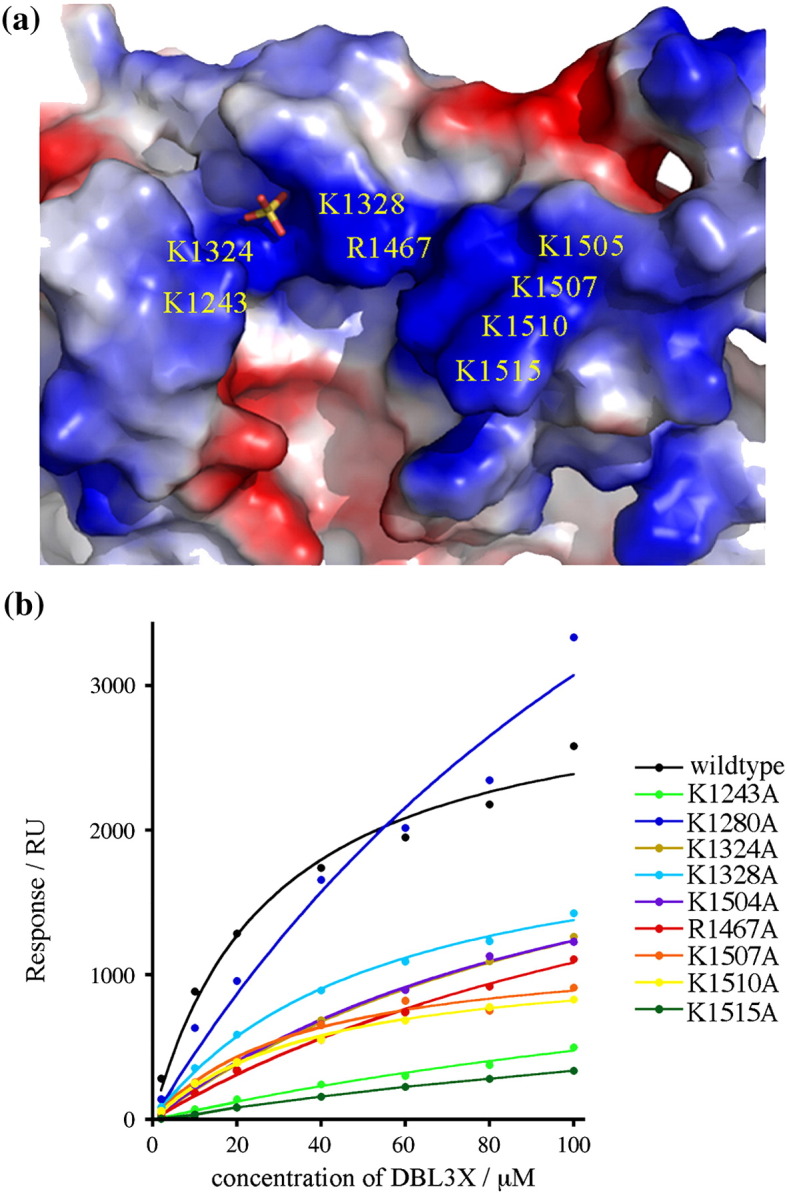
Mutagenesis of the CSPG binding surface of DBL3X. (a) An electrostatic representation of the surface of the DBL3X domain showing the positively charged patch and sulfate-binding site. A bound sulfate ion is shown in stick representation. The side chains that contact the sulfate (K1324 and R1467) are labeled, as are the other side chains that contribute to the positive charge. (b) Plot of the saturated Biacore response obtained against protein concentration for mutants of the DBL3X domain.

Equilibrium analysis of saturated responses due to binding of different concentrations of DBL3X domain to the CSPG-coated surface (Fig. 3b) showed half-maximal binding at a concentration of 33 ± 13 μM. Attempts were also made to fit kinetic data to a variety of binding models, but the dissociation kinetics were complex and did not fit to a simple 1:1 model, suggesting heterogeneity in the interaction between DBL3X and CSPG. This heterogeneity is consistent with the absence of strong, well-defined density in structures soaked with CSA fragments or disaccharides.^32,33^

To map the CSPG-binding site of DBL3X and test predictions from previous structural studies, mutations were made in individual residues that contribute to the sulfate-binding site (K1324A and R1467A) and the positively charged patch (K1243A, K1328A, K1504A, K1507A, K1510A and K1515A) (Fig. 3a). An additional lysine (K1280A) was mutated as a control. While K1280A did not inhibit the interaction, all other mutations reduced binding (Fig. 3b and Table 1). K1243A and K1515A caused the greatest effects with more than 10-fold increases in protein concentrations required for half-maximal binding. These two residues lie at opposite ends of the binding pocket, suggesting that the entire patch plays a role in CSPG binding. Other mutations had a smaller effect, increasing the concentration of protein for half-maximal binding by approximately two to fivefold. Mutations in residues that coordinate the sulfate ion (K1324 and R1467) increased the concentration that gave half-maximal binding by about fourfold.

These studies confirm previous findings that both the positively charged patch and sulfate-binding pocket contribute to the CSPG-binding site of DBL3X.^32,33^ These residues are well conserved in strains of the parasite from different geographical locations and lie in a region of the domain that is surface-exposed in intact VAR2CSA and is targeted by antibodies from multigravid women from endemic areas.^25,37^ This binding site is therefore available for CSPG binding in infected erythrocytes and is available for use as a CSPG-binding site *in vivo*.

### The CSPG binding surface of DBL6ɛ

**Fig. 4:**
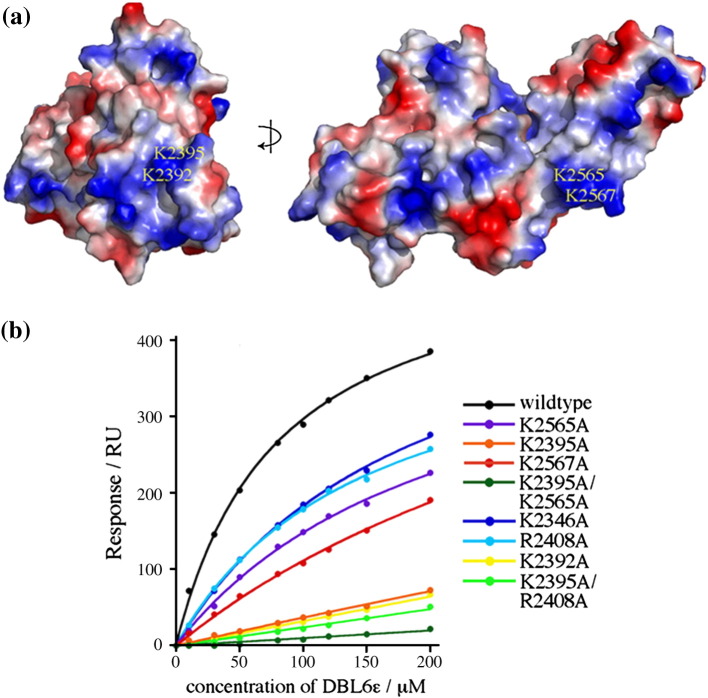
Mutagenesis of the CSPG binding surface of DBL6ɛ. (a) Electrostatic representation of the surface of the DBL3X domain from two perpendicular directions, showing the major positively charged surface patches. (b) Plot of the saturated Biacore response obtained against protein concentration for mutants of the DBL6ɛ domain.

DBL6ɛ lacks both of the features that make up the CSPG-binding surface of DBL3X, with a short loop in the place of the sulfate-binding loop and no corresponding positively charged patch. To confirm that DBL6ɛ is able to bind to CSPG, we used surface plasmon resonance, and showed clear interaction with half-maximal binding at 80 ± 5 μM (Fig. 4b).

We then tested the effect of mutation of different positively charged residues on this binding. The structure showed the presence of two major regions of positive charge (Fig. 4a). The first contains residues K2565 and K2567 and lies on subdomain 3. The second patch consists of residues K2392 and K2395 and lies at the distal side of subdomain 2. We mutated each of these residues to alanine, together with two additional residues not in either of these patches, K2346 and R2408. Of these surface regions, antibody binding studies show that the patch consisting of residues K2392 and K2395 is exposed in VAR2CSA expressed on the surface of infected erythrocytes, while the other mutated residues do not show surface exposure *in vivo*.^37^

Each of these tested mutations reduced CSPG binding (Fig. 4b and Table 2). K2346A, R2408A, K2565A and K2567A each resulted in small decreases in binding, increasing the half-maximal binding concentration by between two- and fivefold. Much more significant effects were seen with K2392A or K2395A. These mutants bound extremely weakly to CSPG, and the concentration required for half-maximal binding could not be estimated at the protein concentrations used. Double mutants containing K2395A together with either R2408A or K2565A bound slightly weaker than K2395A alone.

These studies show that the positively charged patch containing K2392 and K2395 forms the primary CSPG-binding surface of DBL6ɛ. Other residues play a minor role in CSPG binding in this *in vitro* system. Several lines of evidence support the identification of K2392 and K2395 as a CSPG-binding site that is accessible to CSPG *in vivo*. Firstly, while DBL6ɛ is significantly more polymorphic than other domains from VAR2CSA and polymorphisms distribute over the entire surface,^38^ positively charged residues in this binding surface, including K2392 and K2395, are conserved. In addition, the surface patch containing K2392 and K2395 is exposed in intact VAR2CSA and reacts with antibodies from multigravid women.^37^ In contrast, there is little evidence of surface-reactive antibodies against subdomain 3, suggesting that the small reduction in binding due to mutation of residues on this region do not imply that this is part of the CSPG-binding surface *in vivo*.

We have therefore identified a surface–exposed, positively charged patch containing residues K2392 and K2395 as forming the CSPG-binding surface of DBL6ɛ. The surface location of these residues in intact VAR2CSA suggests that this site can also contribute to CSPG binding *in vivo*. The different location and nature of this binding pocket from the residues used by DBL3X to bind to CSPG shows that these two CSPG-binding DBL domains from VAR2CSA do not interact with their ligands through a conserved binding surface.

### Carbohydrate specificity of the DBL domains

Erythrocytes infected with strains taken from placental tissue bind selectively to 4-sulfated chondroitin sulfate, while other glycosaminoglycan carbohydrates do not inhibit binding.^9–11^ In addition, VAR2CSA is selectively precipitated on 4-sulfated chondroitin, but not on other chondroitin species,^23^ suggesting that this protein contains the determinants for specificity. However, crystals of DBL3X soaked with CSA disaccharides show electron density for only the sulfate ion,^32^ revealing no obvious selectivity pocket for other parts of the carbohydrate molecule, and crystals grown in the presence of longer CSA oligosaccharides do not show the presence of well-defined, continuous electron density for ligand.^33^ Crystals of DBL6ɛ grown in the presence of different fragments of chondroitin sulfate also show no ordered ligand. In each case, structural studies do not reveal clear determinants of carbohydrate specificity in single DBL domains.

**Fig. 5:**
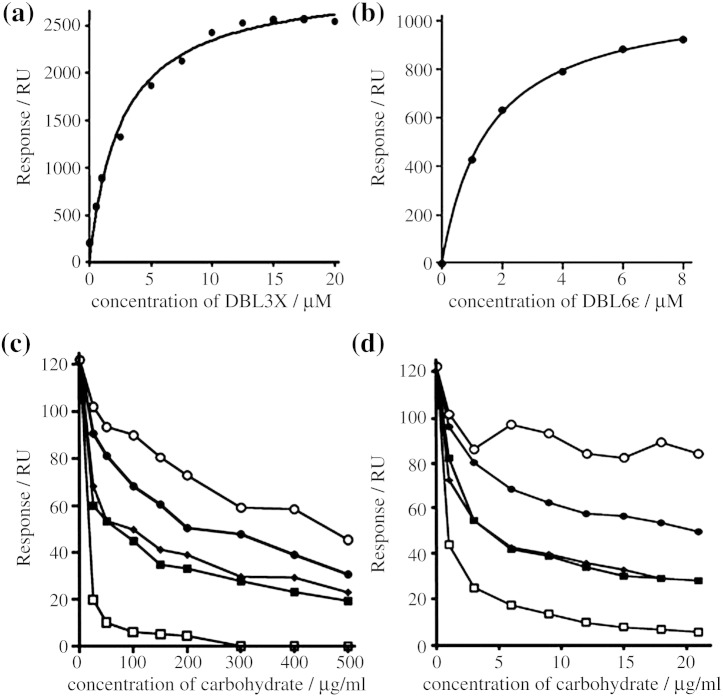
The carbohydrate specificity of the DBL3X and DBL6ɛ domains. (a and b) Plots of the saturated Biacore response against protein concentration for the binding of (a) DBL3X and (b) DBL6ɛ to a heparin-coated surface. (c and d) Competition experiments in which DBL3X (c) and DBL6ɛ (d) were incubated with different concentrations of (●) CSA, (■) dermatan sulfate, (♦) chondroitin sulfate C, (○) hyaluronic acid and (□) heparin before assessment of binding to a CSPG-coated Biacore chip surface. The specific binding to CSPG is shown as an average of three measurements.

To determine whether DBL3X and DBL6ɛ bind specifically to CSA, we coated a surface plasmon resonance chip with porcine intestinal heparin. The domains bound to this surface with half-maximal binding at a concentration of 3.6 ± 0.3 μM for DBL3X (Fig. 5a) and 1.5 ± 0.1 μM for DBL6ɛ (Fig. 5b). Therefore, while heparin does not prevent the binding of infected erythrocytes to a CSPG-coated surface, it does bind to DBL3X and DBL6ɛ, with each domain showing a greater than 10-fold stronger binding to heparin than to CSPG. These results agree with the recently published study by Resende *et al.*,^26^ who also find that individual domains from VAR2CSA, expressed in the baculovirus system, are able to bind to heparin sulfates.

To determine whether heparin and other carbohydrates are able to prevent the binding of DBL3X and DBL6ɛ to CSPG, we tested their ability to compete for protein binding with a CSPG-coated Biacore chip surface. Five different carbohydrates were used in these studies, with carbohydrates obtained from the same sources used to study the inhibition of binding of infected erythrocytes to CSPG.^10^ Hyaluronic acid from human umbilical cord is nonsulfated and consists of alternating d-glucuronic acid and β-*N*-acetylglucosamine, albeit containing approximately 25% contaminating chondroitin sulfates. Chondroitin sulfates A and C consist of alternating β-glucuronic acid and *N*-acetyl-β-galactosamine with sulfates on the 4- and 6-positions of the *N*-acetylgalactosamine, respectively. Dermatan sulfate shows a similar sulfation pattern with alternating iduronic acid and 4-sulfated β-d-acetylgalactosamine. Heparin from porcine intestinal mucosa is more highly sulfated, consisting of 2-O-sulfated iduronic acid and 6-O-sulfated, N-sulfated glucosamine. These five glycosaminoglycan carbohydrates contain differences in sulfation, negative charge and carbohydrate backbone structure.

DBL domains were preincubated with different concentrations of carbohydrates before passing over the CSPG-coated Biacore chip surface and responses were measured. All five carbohydrates inhibited binding of DBL3X and DBL6ɛ, with the greatest inhibition produced by highly sulfated heparin structures (Fig. 5c and d). The least inhibition was observed when unsulfated hyaluronic acid was used, and this might be due to contaminating chondroitin sulfates. The chondroitin sulfates and dermatan sulfate showed an intermediate ability to inhibit binding, and CSA was a weaker inhibitor than chondroitin sulfate C or dermatan sulfate. Therefore, individual DBL domains lack the carbohydrate-binding specificity seen in the interaction of CSPG with infected erythrocytes.

## Discussion

The binding of VAR2CSA on the surface of infected erythrocytes to CSPG in the placenta plays a major part in malaria during pregnancy, and an understanding of this interaction will be valuable in the development of therapeutics to target this form of the disease. In this study, we present the structure of the DBL6ɛ domain of VAR2CSA and characterisation of the CSPG-binding sites of the DBL3X and DBL6ɛ domains.

The DBL6ɛ domain shares the same basic α-helical architecture as that of other DBL domains, with the four α-helices of subdomain 2 and the two long α-helices of subdomain 3 aligning closely to other structures. Upon this structural scaffold there is significant variation in loop length and composition, surface charge properties and disulfide bond locations. It is these differences that lead to the great diversity in binding properties observed for different DBL domains and contribute to variation that facilitates immune evasion. However, even for two domains, DBL3X and DBL6ɛ, which bind to the same ligand, structural comparison reveals significant differences in loop structures and surface charge distribution.

We developed a binding assay based on surface plasmon resonance to study the interaction of DBL domains with CSPG. We show clear binding of DBL3X and DBL6ɛ, while DBL1X and DBL4ɛ do not bind, confirming that CSPG binding is not a universal property of DBL domains from VAR2CSA. We then used mutagenesis to map which parts of the domains form the CSPG-binding surface in each of DBL3X and DBL6ɛ. DBL3X contains a single, large, positively charged patch on the concave surface of the domain that is completed by the folding of a polymorphic loop (residues 1325–1333) upon binding to the sulfate moiety of a carbohydrate.^32^ We confirm that both the sulfate-binding pocket and the positively charged patch contribute to the CSPG-binding site of DBL3X. In contrast, DBL6ɛ lacks both of these surface features, and the CSPG-binding site consists of a surface-exposed, positively charged patch on the distal side of subdomain 2. Both binding sites are formed from residues conserved in parasite isolates from different geographic locations^25,38^ and are surface-exposed in intact infected erythrocytes.^25,37^ Therefore, these two DBL domains, while built on the same α-helical scaffold, both bind to CSPG *in vitro*, but both use very different surface features for this interaction.

A striking characteristic of VAR2CSA-expressing infected erythrocytes is their strong CSPG-binding specificity.^9,10^ While it is possible that this specificity may, in part, be due to interactions with additional unknown receptors, one study has shown that isolated VAR2CSA protein does show carbohydrate specificity, as it can be precipitated with CSA but not CSC.^23^ In contrast, DBL3X and DBL6ɛ do not contain the determinants for selection of CSPG, but bind more effectively to carbohydrates with increased charge and sulfation. Indeed, structures of DBL3X and DBL6ɛ show no clear binding pocket that could account for this specificity, and cocrystals reveal no well-ordered ligands. This raises questions about whether the CSPG-binding surfaces that we have identified on DBL3X and DBL6ɛ, using our *in vitro* assay, contribute to ligand binding *in vivo*. Indeed, DBL domains from PfEMP1 proteins that are not upregulated in placental strains can bind to CSPG and other carbohydrates in isolation, raising fear of false positives.^26^ Nevertheless, VAR2CSA is the PfEMP1 upregulated in placental malaria, showing that this is the physiologically relevant CSPG receptor. In addition, antibody depletion studies have been used to map which parts are exposed on infected erythrocytes, showing that the surfaces that we have identified as CSPG-binding sites on DBL3X and DBL6ɛ are both exposed on the surface of infected erythrocytes and are therefore available for CSPG binding *in vivo*.^37^ We have therefore shown that DBL3X and DBL6ɛ, although determined by crystallography to adopt the correct DBL domain fold, do not bind specifically to CSPG. In addition, a recent study in which each of the DBL domains from VAR2CSA were expressed using the baculovirus expression system confirms these findings and shows that no other domains from VAR2CSA bind specifically to CSPG.^26^ Therefore, individual DBL domains from VAR2CSA do not, alone, confer the CSPG-binding specificity of intact erythrocytes.

These data combine to suggest that VAR2CSA does not consist of a series of independent, specific, CSPG-binding domains that interact with their ligand through a conserved binding site. Instead individual domains provide distinct surface-exposed patches that contribute different features to CSPG binding. These positively charged patches and sulfate-binding sites, contributed by different domains, are surface-exposed on the infected erythrocyte and are available for CSPG binding *in vivo*. How these domains come together to generate the specific CSPG-binding site observed in VAR2CSA is uncertain. However, several pieces of evidence suggest that VAR2CSA does not consist of a series of DBL domains linked through flexible linkers, but that it adopts higher-order structure. Large parts of VAR2CSA, including the linkers between DBL domains, are not polymorphic in isolates from pregnant women from different geographical locations.^25,37^ This suggests that they are not under selection pressure to vary, as would be expected if exposed to the immune system. In addition, only restricted parts of the domain surfaces are accessible to antibodies from pregnant women.^37^ This all leads to a model in which the domains of VAR2CSA are organised in three-dimensions, positioning different surfaces involved in CSPG binding to form a specific binding pocket. A full understanding of the binding mechanism of VAR2CSA for CSPG will therefore require structures of larger fragments of VAR2CSA showing how these domains fit together. Nevertheless, antibodies raised against isolated domains, including DBL6ɛ, have been shown to inhibit binding of infected erythrocytes to CSPG,^30,31^ suggesting that therapeutics targeting a single domain can be effective. Therefore, information about the exposed binding surfaces contributed by DBL3X and DBL6ɛ to CSPG binding can provide valuable guidance for rational development of therapeutics.

## Materials and Methods

### Expression, mutagenesis and purification of the DBL3X domain

The DBL3X domain of VAR2CSA (accession codes AY372123 and AAQ73926) was cloned from A4 strain genomic DNA with a six-histidine residue tag at the N-terminus as described in Ref. 39. Single point mutations were made using a polymerase chain reaction-based mutagenesis protocol. The forward primers were K1243A (AAAATATTTCCAG GTGCAGGAG GCGAGAAACAA), K1280A (AGTTATGGG ATGCAAGTTATGGTGGAA), K1324A (CAGCAATTATATCAGCAAATGATAAAAAAGG), K1328A (CAAAAAATGATAAAGCA GGACAAAAAGGAAAA), R1467A (GTATAGAACGATTA GCATATGAACAAAATATA), K1504A (GGAGCATGTAAAAGAGCATGTGAAAAATATAA), K1507A (AAAAGAAAAT GTGAAGCATATAAAAAATATATT), K1510A (GTGAAAAATATAAAGCATATATTTCT GAAA) and K1515A (AATATATTTCTGAAGCAAAACAAGAATGGGA). Reverse primers were the reverse complement of the forward primers. Mutagenesis was carried out as described for the Quikchange mutagenesis method (Stratagene) and plasmids were checked by DNA sequencing. Protein was expressed and purified as described in Ref. 39, and circular dichroism measurements were used to confirm that mutations had not altered the structure of the domain.

### Cloning, expression and purification of the DBL1X, DBL4ɛ and DBL6ɛ domains

The DBL1X (residues 81–428, accession code AY372123), DBL4ɛ (residues 1595–1909, accession code AY372123) and DBL6ɛ (residues 2333–2634, accession code AE014844.1) domains of VAR2CSA were cloned from genomic DNA and ligated into the BamHI–NheI site of a modified pEt15b plasmid (Novagen) in-frame with the tobacco etch virus (TEV) cleavage site.^39^

The C2480S mutant of DBL6ɛ was made using a polymerase chain reaction-based mutagenesis protocol using forward primer TGGG AATCTATGTTATCTGGATACAAACATGCC and the reverse complement. Mutagenesis was carried out as described for the Quikchange mutagenesis method (Stratagene) and confirmed by DNA sequencing.

The plasmids were transformed into Origami B *Escherichia coli* (Novagen) containing the pRIG plasmid.^40^ The cells were grown in 2xYT medium at 37 °C to reach an optical density of 1.4 at 600 nm and were then induced with 1 mM IPTG. The expression of the soluble His-tagged protein was allowed to take place overnight at 25 °C.

Cells were harvested by centrifuge and resuspended in buffer 1 [20 mM Tris (pH 8.0), 0.3 M NaCl, 10 mM imidazole, 0.5% Triton X-100] and lysed by sonication. The cell lysate was centrifuged for 30 min at 45,000*g* and purified by affinity chromatography using Ni–NTA Sepharose (Qiagen). The supernatant was loaded onto a Ni–NTA affinity column, washed with buffer 1 and buffer 2 [20 mM Tris (pH 8.0), 0.5 M NaCl, 10 mM imidazole] and eluted with 20 mM Tris (pH 8.0), 0.1 M NaCl and 0.2 M imidazole.

Buffer exchange of the protein into 20 mM phosphate (pH 7.4), 150 mM NaCl, 3 mM reduced glutathione and 0.3 mM oxidized glutathione was done prior to the addition of 1 mg of TEV protease per 10 mg of protein. The cleavage reaction was incubated overnight at room temperature. Cleaved protein was passed through a Ni–NTA affinity column (to remove uncleaved protein and TEV protease), followed by a Q-Sepharose column (GE Healthcare) and gel filtration using a Superdex 200 16/60 column (GE Healthcare) with buffer [20 mM Tris (pH 8.0), 50 mM NaCl]. The protein was concentrated to 10 mg/ml with an Amicon Ultra centrifugal filter device (10,000 Da molecular mass cutoff). Each litre of bacterial culture yielded 2.5–5.0 mg of purified protein.

### Mutagenesis of DBL6ɛ

Site-directed mutagenesis was performed with a polymerase chain reaction-based mutagenesis protocol using primers K2346A (GTTAATATGAAAGCAAATAATGATGATA), K2392 (AAAAGAGATCCTGCATTGTTTAAAGAT), K2395 (CCTAAATTGTTTGCAGATTTCA TTTAT), K2408A (CTGAAGTTGAAGCGTTAAAAAAAGTATATG), K2565A (TTTATT TTAATAGCAAAAAAGGAGTATC) and K2567A (TTAATAAAAAAAGCGGAGTATCA GTCAC). Reverse primers were the reverse complement of the forward primers. Mutagenesis was carried out as described for the Quikchange mutagenesis method (Stratagene). Plasmids were checked by DNA sequencing and protein was expressed and purified as above. Double mutations were carried out sequentially.

### Production of selenomethionine-labeled DBL6ɛ domain

Cells initially grown in 2xYT medium to an optical density of 1.4 at 600 nm and pelleted before resuspension in M9 media (20% glucose, 40 mM Na_2_HPO_4_, 20 mM KH_2_PO_4_, 20 mM NH4Cl, 8.5 mM NaCl, 1 mM MgSO_4_, 100 mg/l each of l-lysine, l-phenylalanine and l-threonine and 50 mg/l each of l-isoleucine, l-leucine, l-valine and l-selenomethionine). They were incubated at 37 °C for a further 1.5 h, induced with 1 mM IPTG and incubated overnight at 25 °C to allow expression to take place. The selenomethionine-labeled protein was purified as above.

### Crystallisation of the DBL6ɛ domain

Crystals were grown using the hanging-drop vapour-diffusion method by mixing 1 μl of protein solution with 1 μl of reservoir solution and 0.5 μl of additive solution (0.01 M glutathione reduced, 0.01 M glutathione oxidized) and equilibrating against 1 ml of reservoir solution. Crystals grew in 18–28% polyethylene glycol (PEG) 4000, 0.1 M Tris (pH 6.0–8.0) and 0.1–0.7 M sodium acetate. They were visible after 4–6 days and continued to grow for 10–14 days. Crystals of selenomethionine-labeled protein were grown in the same condition as the native and appeared within 4–6 days. For cocrystallisation, CSA fragments were prepared as described in Ref. 32. CSA fragments (2mer, 8mer and 12mer) were incubated with the DBL6ɛ domain protein with molar ratios of 1:10 for 2 h at room temperature prior to setting up droplets. Crystals from cocrystallisation formed in the same conditions as native crystals.

### Data collection and structure determination of the DBL6ɛ domain

The selenomethionine-labeled crystals for multiple-wavelength anomalous dispersion (MAD) experiments were grown in 26% PEG 4000, 0.1 M Tris (pH 8.0) and 0.5 M sodium acetate. They were cryoprotected by transfer into 25% glycerol, 34% PEG 4000, 0.1 M Tris (pH 8.0), 0.5 M sodium acetate, 200 mM ascorbate and 0.5 mM β-mercaptoethanol and flash-frozen in liquid nitrogen. Diffraction data were collected to 3.0 Å at 100 K on beamline BM14 at the European Synchrotron Radiation Facility (ESRF, Grenoble, France). Native data sets were also collected at 100 K on beamline I02 at the Diamond Light Source using crystals grown in 30% PEG 4000, 0.1 M Tris (pH 8.0) and 0.35 M sodium acetate.

Native and MAD data were processed using MOSFLM^41^ and SCALA^42^ from the CCP4 suite^43^ and were consistent with a primitive tetragonal lattice. Systematic absences in the 00l and *h*00, 0l0 reflections indicated that the crystals belong to space group *P*43212. The structure was determined using MAD phasing with data from a selenium derivative. The asymmetric unit of the crystal contained two molecules, and 18 Se sites were found and refined using autoSHARP.^44^ MAD phases, following density modification, were used to calculate an initial map that was used for model building in Coot.^45^ The structure was refined against native data (100° of data at 3.2 Å) using TLS refinement in Refmac5^46^ with 5% reflections kept excluded for *R*_free_ evaluation. Data processing and structure refinement statistics are outlined in Tables 3 and 4.

### Surface plasmon resonance measurements

Measurements were performed on a Biacore 2000 instrument with a constant flow rate of 30 μl/min. Placental CSPG was obtained from MR4 (deposited by C. Gowda) and coupled to the surface of a CM5 chip (Biacore) using the amine coupling protocol as recommended by the manufacturer. Briefly, channels 1 and 2 were treated with a 1:1 mixture of 1-ethyl-3-(3-dimethylaminopropyl) carbodiimide and *N*-hydroxysuccinimide (Biacore). Placental CSPG was dissolved to a concentration of 100 μg/ml in 10 mM sodium acetate (pH 5.0) and flowed through channel 2 until the response increased by 400 RU. The remaining reactive groups on both surfaces were then blocked with ethanolamine.

Proteins were equilibrated using PD10 columns (Amersham Biosciences) into HBS buffer [10 mM Hepes (pH 7.4), 150 mM NaCl, 50 μM ethylenediaminetetraacetic acid, 0.05% Tween 20] and concentrated for Biacore analysis. Both channels were equilibrated with HBS buffer before injection of purified DBL domain protein. The level of specific binding was obtained from a subtraction of the response from channel 2 from that of channel 1. After each injection, both channels were regenerated with a 30-μl injection of 5 mM NaOH, followed by a 30-μl injection of 5 M NaCl. This procedure led to recovery of the original baseline and regenerated a CSPG surface that could bind reproducibly to subsequent injections of DBL3X protein.

Data were analysed using the BIAevaluation software to obtain maximum response values for saturated curves. These were plotted against the concentration of protein using Prism 5.01 (GraphPad Software Inc., CA) and fitted to a single-site binding model, allowing determination of the concentration that gave half-maximal response. Errors are the given values with 95% confidence. The BIAevaluation software was used to analyse the kinetics of association and dissociation.

### Carbohydrate competition experiments

Carbohydrates were obtained from Sigma: CSA from bovine trachea (C9819), chondrotin sulfate C from shark cartilage (C4384), dermatan sulfate from porcine intestinal mucosa (C3788), heparin from porcine intestinal mucosa (H9399) and hyaluronic acid from human umbilical cord (53750). Each carbohydrate was dissolved in HBS to 1 mg/ml and filtered through a 0.2-μm membrane (Sartorius).

These stocks were mixed with DBL domain to produce samples with a final concentration of 5 μM for DBL3X or 15 μM for DBL6ɛ and a range of carbohydrate concentrations (25–500 mM) in HBS. These were incubated for a minimum of 30 min before Biacore measurements were taken as described above.

### Heparin binding studies

Porcine intestinal heparin was dissolved to 5 mg/ml in 20 mM sodium phosphate (pH 7.4) and 150 mM NaCl. One milliliter of this heparin solution was incubated with 25 μl of 50 mM biotin-hydrazide (Pierce) and 15 μl of 1-ethyl-3-(3-dimethylaminopropyl) (100 mg/ml) for 2 h at 25°C. The heparin was then dialysed overnight into 20 mM sodium phosphate (pH 7.4) and 150 mM NaCl and 100 μl was injected over flow cell 2 of a streptavidin-coated SA chip (Biacore).

Channels 1 and 2 of this chip were equilibrated with HBS buffer before injection of purified DBL3X or DBL6ɛ domain. The level of specific binding was obtained from a subtraction of the response from channel 2 from that of channel 1. After each injection, both channels were regenerated with a 30-μl injection of 2 M NaCl, restoring the signal to original levels. Data were analysed as above.

### PDB accession numbers

Coordinates and structure factors have been deposited in the PDB with accession number 2wau.

## Figures and Tables

**Table 1:** Analysis of the binding of DBL3X and mutants to CSPG

	Concentration of DBL3X that gives half-maximal binding (μM)
Wild type	33 ± 13
K1243A	367 ± 50
K1324A	122 ± 24
K1328A	89 ± 22
R1467A	122 ± 12
K1504A	102 ± 14
K1507A	172 ± 83
K1510A	193 ± 67
K1515A	488 ± 38

**Table 2:** Analysis of the binding of DBL6ɛ and mutants to CSPG

	Concentration of DBL6ɛ that gives half-maximal binding (μM)
Wild type	80 ± 5
K2346A	190 ± 10
K2392A	Not determined
K2395A	Not determined
R2408A	151 ± 9
K2565A	215 ± 21
K2567A	440 ± 60
K2395AR2408A	Not determined
K2395AK2565A	Not determined

**Table 3:** Data collection statistics

	Se-DBL6ɛ			Native DBL6ɛ
	Peak	Edge	Remote	
Beamline	BM14 ESRF			I02 Diamond
Space group	*P*4_3_2_1_2			*P*4_3_2_1_2
Unit cell parameter (Å)	*a*, *b* = 63.76, *c* = 333.48			*a*, *b* = 62.94, *c* = 334.27
Resolution limit (Å)	83.33–3.0			111.11–3.0
Wavelength (Å)	0.97855	0.97871	0.91999	0.9200
*R*_mrg_ (%)	8.2 (48.0)	9.6 (64.1)	10.9 (61.4)	10.2 (60.2)
*I*/σ(*I*)	16.0 (3.9)	24.4 (5.2)	25.1 (4.9)	14.2 (2.9)
Completeness (%)	99.5 (99.4)	99.5 (99.4)	99.5 (99.7)	99.9 (100)
Multiplicity	6.6 (6.8)	6.5 (6.8)	6.5 (6.8)	5.8 (6.2)
Anomalous completeness	99.9 (99.6)	99.9 (99.7)	99.4 (99.9)	
Anomalous multiplicity	3.7 (3.6)	3.7 (3.6)	3.6 (3.7)	

**Table 4:** X-ray refinement statistics of DBL6ɛ

RMSD bond lengths (Å)	0.069
RMSD bond angles (°)	0.893
Reflection used for refinement (work/free)	14481/2038
*R*_work_ (%)	28.9
*R*_free_ (%)	32.5
No. of protein residues	584
Ramachandran plot	
Allowed region (%)	83.6
Additional allowed region (%)	16.4
Generously allowed region (%)	0
Disallowed region (%)	0
